# Psychrotrophic *Pseudomonas mandelii* CBS-1 produces high levels of poly-β-hydroxybutyrate

**DOI:** 10.1186/2193-1801-2-335

**Published:** 2013-07-23

**Authors:** Rongpeng Li, Yuji Jiang, Xinfeng Wang, Jingjing Yang, Yuan Gao, Xiaoli Zi, Xia Zhang, Haofeng Gao, Nan Hu

**Affiliations:** Colloge of Biotechnology and Pharmaceutical Engineering, Nanjing University of Technology, Nanjing, 211800 People’s Republic of China; Institute of Soil Science, Chinese Academy of Sciences, Nanjing, 210008 People’s Republic of China; Jiangsu Key Laboratory for Biomass-based Energy and Enzyme Technology, Huaian, 223300 People’s Republic of China

**Keywords:** PHB, *Pseudomonas mandelii* CBS-1, Facultative psychrotrophs, ^1^H- nuclear magnetic resonance, Fermentation

## Abstract

A novel facultative psychrotroph (strain CBS-1), which accumulates poly-β-hydroxybutyrate (PHB), was isolated from soil samples taken from Changbai Mountain, China. Phylogenetic analysis based on 16S rRNA sequence data and Biolog analysis identified strain CBS-1 as *Pseudomonas mandelii*. Transmission electron micrographs revealed abundant electron-transparent intracellular granules. ^1^H-nuclear magnetic resonance analysis revealed that the granules were composed of PHB. *P*. *mandelii* CBS-1 grew optimally at 20°C. When cultured aerobically for 48 h with sucrose as the sole carbon source, strain CBS-1 yielded a maximum cell density of 29.3 g/L cell dry weight and synthesized 22.3 g/L of PHB. The ability of strain CBS-1 to grow at a low temperature and rapidly synthesize high levels of PHB may reduce the costs of industrial PHB production.

## Background

Polyhydroxyalkanoates (PHAs) are biodegradable polyesters composed of (R)-3HA monomers, which are ubiquitously synthesized by microorganisms. Many bacteria store carbon as PHAs to protect themselves from nutrient limitation, including lack of nitrogen, phosphorus, and magnesium (Dowes and Senior, 1973). PHAs are synthesized by most genera of Bacteria and (Archaea Steinuchel and Byrom, 1991). The best-characterized microorganisms that produce PHAs are *Alcaligenes* sp. (Linko et al., 1993 sp. *Azotobacter* (Page,^1992^), *Bacillus* sp. (Huang and Reusch, 1996), *Rhodopseudomonas* sp. (Yue et al., 2007), and *Pseudomonas* sp. (Kimura et al., 1992).

Poly-β-hydroxybutyrate (PHB), the least complex PHA biopolyester, offers distinct advantages for polyester production because of its biodegradability, lighter mass, thermoplasticity, and resistance to abrasion (Steinbuchel and Valeniin, 1995). PHB shows great potential for manufacturing products such as degradable plastics, medical implant materials, and fine chemicals (Holemes, 1985). Unfortunately, available bacterial strains synthesize low levels of PHB even after prolonged fermentation. Therefore, PHB biosynthesis is more expensive than standard chemical synthesis and limits its use (Choi and Lee, 1999).

Therefore, the goal of our research was to isolate bacteria capable of producing high levels of PHB at low temperatures. We report here the isolation of a facultative psychrotrophic strain (CBS-1) from the soil of Changbai Mountain, China, and its identification as *Pseudomonas mandelii*. It is the first report of PHB accumulation in *P*. *mandelii*. Strain CBS-1 grew aerobically to a dry-cell weight of 29.3 g/L and synthesized 22.3 g/L of PHB in 48 h, which is higher compared with other reported strains (Kimura et al., 1992; Huang and Reusch, 1996; Yue et al., 2007).

## Material and methods

### Strains, growth media, and culture condition

A soil sample from Changbai Mountain (altitude 2183 m, N 42°08′19.7″, E 128°10′8.3″), Jilin Province, China, was suspended in sterile H_2_O, vortexed thoroughly, and cultured in an enriched medium containing 5 g/L yeast extract, 10 g/L tryptone, and 2.5 g/L NaCl at 20°C for 2 days. A 50-μL aliquot was spread on PHB detection agar (20 g/L glucose, 2 g/L (NH_4_)_2_SO_4_, 13.3 g/L KH_2_PO_4_, 1.2 g/L MgSO_4_^.^7H_2_O, 1.7 g/L citric acid, 1.7 g/L trace elements solution, 15 g/L agar, and 0.5 μg/mL Nile blue A (Spiekermann et al., 1999). After overnight incubation at 20°C, plates were observed under ultraviolet (UV) light. Fluorescent colonies were purified by streaking on the same agar plates, and the isolates were further characterized by staining with Sudan Black B (Lee and Choi, 2004).

### Morphological characteristics

Cell morphology was examined using transmission electron microscopy as follows: a 2-mL aliquot of cells in late exponential phase (optical density OD_600_ 55–80) was fixed with 4% glutaraldehyde and 1% osmium tetroxide and dehydrated by sequential treatment with 30%, 50%, 70%, 80%, 90%, and 100% acetone. The dehydrated cells were immersed in epoxy resin and then transferred into sample boats. The resin was polymerized for 24 h at 30, 45, and 60°C. Ultra-thin sections were prepared using an ultramicrotome, stained sequentially with uranyl acetate and lead citrate for 22 min and 5 min, respectively, and observed using an H-7650 transmission electron microscope (Hitachi, Tokyo, Japan).

### Biolog Gram-negative (GN) assay

Gram staining was performed as described by Magee et al. (1975). A pure culture was grown on a BUG agar plate (Biolog Catalog #70101). Biolog™ GN 96-well microtiter plates contain 95 different carbon sources, a negative control, and a tetrazolium dye (Biolog Inc., Hayward, CA, USA). Cells were swabbed from the surface of the plate and suspended to a specific density in GN Inoculating Fluid (Biolog Catalog #72101). The well of the Biolog GN2 MicroPlate (Biolog Catalog #1011) was inoculated individually with 150 μL of diluted suspensions and then incubated at 30°C for 24 h (Garland and Mills, 1991). The MicroPlate was inspected either visually or analyzed using the Biolog MicroStation™ and the results compared with the GN III Database according to the manufacturer’s instructions (Biolog Catalog #22730D).

### Polymerase chain reaction (PCR) amplification of the 16S rRNA gene

The 16S rRNA gene was amplified using PCR with the universal bacterial primers 27F (5′-agagttgatcctggctcag-3′) and 1492R (5′-ggytaccttgttacgactt-3′) (Lane, 1991), and the product was sequenced by Takara Corp. (Dalian, China). The sequence was compared with 16S rRNA gene sequences in the GenBank database (http://www.ncbi.nlm.nih.gov) using the BLAST algorithm (Altschul et al., 1990). A neighbor-joining tree was constructed using the MEGA 4.0 program (Kumar et al., 2004).

### Poly-β-hydroxybutyrate extraction

Cells were harvested and treated with 10% sodium dodecyl sulfate at 100°C for 15 min. After centrifugation at 10,000 rpm for 20 min, the pellets were washed twice with H_2_O, dried at 40°C, and extracted with chloroform for 1 h at 60°C. Insoluble material was removed by filtration, and the soluble PHB was separated from the chloroform phase by evaporation, washed twice with methanol, filtered, dried at 60–70°C, and weighed using an electronic balance (Sartorius, BSA224S).

### Nuclear magnetic resonance (NMR) spectroscopy

The NMR spectra of 1-mL samples were recorded using a BRUAK AV-400 spectrometer with a 5-mm ^1^H-probe, and deuterated chloroform (CDCl_3_) was used as a solvent at a final concentration of 10 g/L. The ^1^H-NMR spectrum for PHB was recorded at 400 MHz. A PHB standard was purchased from Sigma-Aldrich (St. Louis, MO, USA, #MSDS 363502).

### Effect of culture conditions on cell growth

The effects on cell growth of carbon and nitrogen sources, C/N ratio, temperature, initial pH, and NaCl concentration were investigated using single-factor tests (Tang and Luo, 2008). Specifically, yeast extract, glucose, sucrose, fructose, glycerol, and ethanol were used as carbon source. Nitrogen sources included tryptone, peptone, beef extract, monosodium glutamate, urea, and NH_4_Cl. All media contained 1.0 g/L KH_2_PO_4_, 2.5 g/L Na_2_HPO_4_, pH 7.0, and cells were cultured at 20°C, 200 rpm. To test the effect of the C/N ratio, sucrose and monosodium glutamate served as the sole carbon and nitrogen sources and were added at the C/N ratios as follows: 1, 5, 10, 15, 20, 25, and 30. Other conditions were the same as those described above. The optimum C/N ratio was used to test the conditions described next. NaCl concentrations were varied from 0–50 g/L (5-g/L intervals), and at initial pH values from 5.0 to 9.0 (0.5 pH unit intervals). Cultures were incubated from 5°C to 40°C (5°C intervals). After 3 days, cells were collected by centrifugation, and the pellets were dried at 70°C to a constant weight.

### PHB production by aerated cultures

Cells was cultured at 20°C in medium containing 1.0 g/L KH_2_PO_4_, 2.5 g/L Na_2_HPO_4_, with optimal 1% (w/v) sucrose as the carbon source and monosodium glutamate as the nitrogen source. The pH was adjusted to 7.0 with 0.5 M NaOH. A 10-mL inoculum from a CBS-1 starter culture (OD_600_ 0.5) was added to a 150-mL minimal medium. The 250-mL flasks were incubated at 20°C for at least 3 days and shaken at 200 rpm. Samples were collected at 8-h intervals from 0–72 h, centrifuged, and dried at 70°C to a constant weight.

### Statistical analysis

All results are shown as the average and standard deviation of at least three independent experiments. Student’s *t*-test, analysis of variance (ANOVA), and Duncan’s multiple range test were used to determine the statistical significance of differences in the nitrite-to-nitrogen conversion rate. Statistical significance was defined as *P* < 0.05. All analyses were performed using SPSS for Windows version 11.0.

## Results

### Isolation and characterization of strain CBS-1

**Figure 1:**
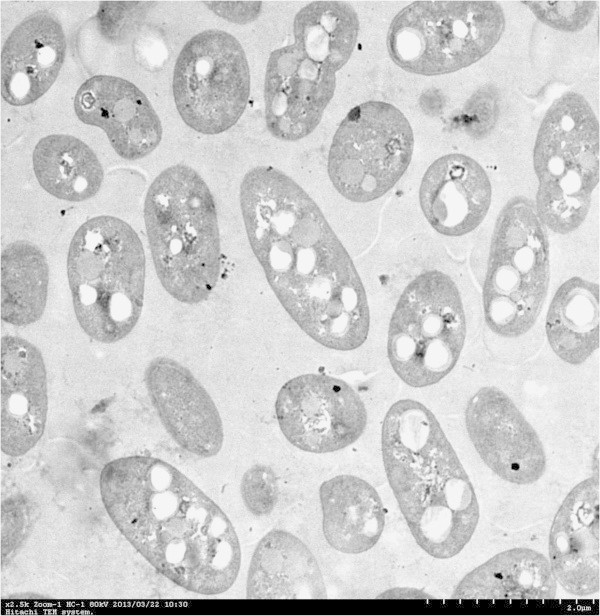
**Transmission electron micrograph of a thin section showing dividing*P. mandelii*CBS-1-containing granules of PHB.** Magnification 2,500×.

Oxazine dye Nile blue A was used to develop a simple and highly sensitive staining method to directly detect poly-3-hydroxybutyric acid and other PHAs in growing bacterial colonies (Spiekermann et al., 1999). A 50-μl soil sample from Changbai Mountain, Jilin Province, China, was spread on PHB-detection agar containing Nile blue A stain (Lee and Choi, 2004). After incubation and observation under UV light, 59 fluorescent colonies were purified, and 27 were stained by Sudan Black B. A clone isolated from the largest and most intensely stained colony was named strain CBS-1. After incubation for 2 days on LB plates, CBS-1 formed white, circular colonies of semitransparent slabs that were wet with smooth surfaces. The bacilliform cells were GN, 3–4 mm in diameter × 5–7 mm long, and did not form spores. The cells occurred singly or in clusters and contained large granules of PHB (Figure 1).

**Figure 2:**
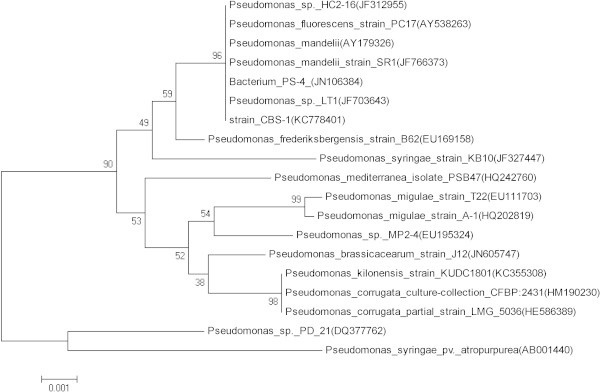
**Phylogenetic tree of*P. mandelii*CBS-1 and other related bacterial genera.** Bootstrap values (%) from 1,000 replicates are as shown. The scale bar represents 0.01 nucleotide substitution per position. Numbers in brackets are GenBank accession numbers for the 16S rRNA sequences.

A stretch of 1335 nucleotides of the 16S rRNA gene was PCR amplified and sequenced. The sequence was submitted to GenBank under the accession number KC778401. BLAST analysis indicated that strain CBS-1 is most closely related (99% identity) to *P*. *mandelii*. Phylogenetic analysis also grouped CBS-1 together with *P*. *mandelii* with 96% bootstrap support (Figure 2).

The GN III MicroPlate Database (Biolog Catalog #22730D) identifies 564 common species in accordance with classical identification methods and current taxonomic nomenclature. Unfortunately, *P*. *mandelii* was not present in the database. The GN MicroPlate performance characteristics indicated that strain CBS-1 was similar to *P*. *fluorescens* biovar C. The similarity index was 0.81 at 24 h (Table 1). *P*. *mandelii* is classified in the *P*. *fluorescens* species (Anzai et al., 2000). Therefore, the Biolog GN assay result did not contradict the 16S rRNA gene sequence, and we concluded that CBS-1 is a *P*. *mandelii* strain.

**Table 1:** **Biochemical characteristics of strain CBS-1 and related*Pseudomonas*species**

Growth on:	Strain CBS-1	***P***. ***fluorescens*** biovar C^a^	***P***. ***fluorescens*** A2a5^b^
L-Arabinose	+	+	+
α-L-Rhamnose	-	-	-
Sucrose	+	+	+
Sebacic acid	-	-	-
Succinic acid	-	-	+
L-Asparagine	+	+	+
L-Aspartic acid	+	+	+
L-Histidine	+	+	+
L-Leucine	+	+	+
L-proline	+	+	+
L-Theronine	+	+	+

+ Positive; -, negative.

^a^Biolog GN assay data.

^b^Data from Jiang et al., (2008).

### Physiological characteristics of CBS-1

**Figure 3:**
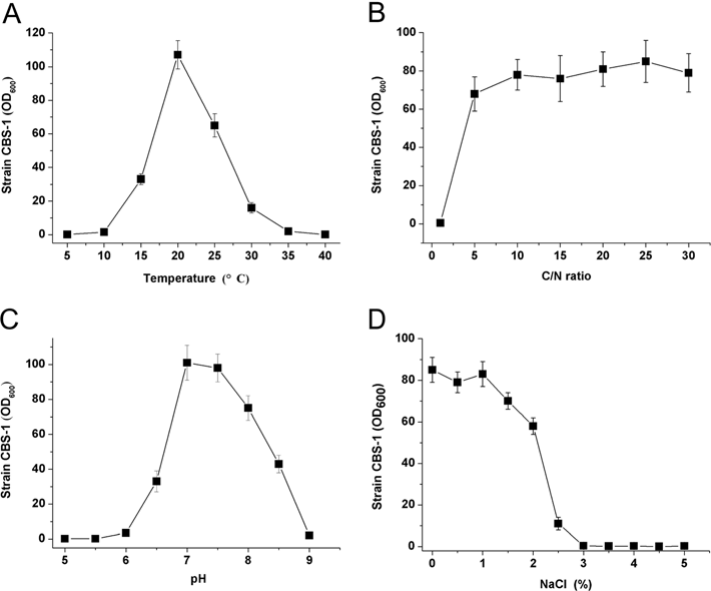
**Effect of culture conditions on*P. mandelii*CBS-1 growth.** Effects of temperature **(A)**, C/N ratio **(B)**, pH **(C)**, and NaCl concentration **(D)** were investigated.

Sucrose and monosodium glutamate were the optimum carbon and nitrogen sources for culturing CBS-1 (Table 2) at a C/N ratio of 5 (Figure 3B). Strain CBS-1 grew to significant numbers between 10 and 35°C, and the optimum growth temperature was 20°C (Figure 3A). Strain CBS-1 grew between pH 6.0 and 9.0 and optimally between pH 7.0 and 7.5 (Figure 3C). Significant growth was observed in media containing 0–3% NaCl (Figure 3D).

**Table 2:** **Effect of different carbon and nitrogen sources on PHB synthesis by strain CBS-1**

Carbon sources	Cell dry weight (g/L)	Nitrogen source	Cell dry weight (g/L)
Yeast extract	30.2 ± 3.1	Tryptone	19.0 ± 0.9
Glucose	9.9 ± 1.4	Peptone	17.4 ± 1.8
Sucrose	29.1 ± 3.0	Beef extract	18.4 ± 1.3
Fructose	7.8 ± 1.1	Monosodium glutamate	23.5 ± 1.0
Glycerol	16.3 ± 2.9	Urea	11.5 ± 0.8
Ethanol	2.9 ± 0.2	NH_4_Cl	13.6 ± 1.5

### PHB synthesis by CBS-1

**Figure 4:**
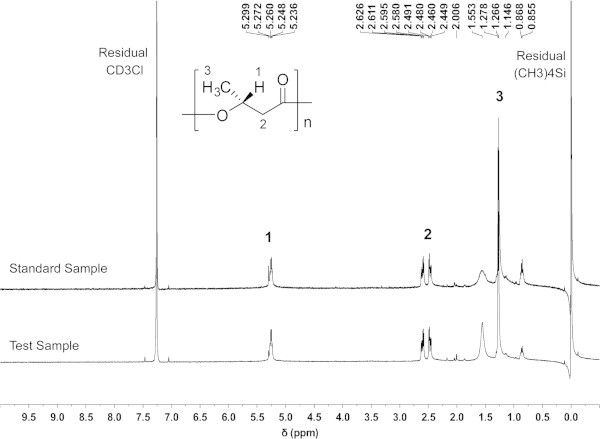
^**1**^**H-NMR spectra of a PHB standard and the PHB produced by*P. mandelii*CBS-1.**

After extracting cells with SDS and isolating PHB as a dried residue as described in Materials and methods, we conducted ^1^H-NMR analysis (Figure 4). PHB peaks were detected at δ = 5.2, 2.5, and 1.2 ppm, which correspond to a –CH doublet, a –CH_2_ multiplet, and a –CH_3_ doublet, respectively (Verlinden et al., 2011; De Rooy et al., 2007). The large peak at δ = 7.3 ppm represents the solvent (CD_3_Cl), and the peaks at δ = 0.0 ppm and δ = 0.8 ppm represent the internal standard (CH_3_)_4_Si. A small peak at δ = 1.6 ppm was attributed to minor contamination of the solvent with H_2_O. These results were identical to those for the PHB standard. Therefore, we concluded that the only PHA synthesized by strain CBS-1 grown in the presence of sucrose as the carbon source was PHB.

### PHB production in aerated CBS-1 cultures

**Figure 5:**
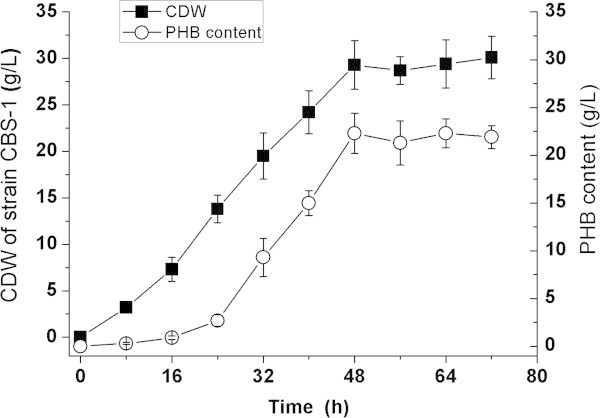
**Aerobic growth of*P. mandelii*CBS-1 and PHB production at 20°C in a medium containing sucrose as carbon source.** Each point represents the average of three cultures.

Strain CBS-1 was cultured in 150-mL sucrose-containing medium, pH 7.0, at 20°C in a 250-mL flask, which was shaken as described in Materials and methods. Cells were harvested upon reaching the stationary phase (48 h). The CDW was 29.3 g/L, and the PHB concentration reached 22.3 g/L (Figure 5). The concentrations of PHB were 9 g/L and 13 g/L when the cells were grown at 15°C and 25°C, respectively, under the same conditions. These data indicate that the yields of PHB depend critically on growth temperature.

## Discussion

Because of the efficient and diverse biosynthetic capabilities of psychrotrophs at relatively low temperatures, these organisms provide an important resource for biotechnology (Moyer and Morita, 2007). Soil samples acquired at circumpolar latitudes are rich in psychrotrophs. For example, Jiang et al. (2008) first isolated the *P*. *fluorescens* strain A2a5 from Alaskan soil, and the *P*. *extremaustralis* sp. nov. strain 14-3^T^ was isolated from an ephemeral pond in Antarctica (Lopez et al., 2009). These two psychrotrophs accumulate large amounts of PHB at a low temperature, strain A2a5 grew at 25°C, while strain 14-3^T^ could grow at a temperature range from 4 to 37°C. The nucleotide sequence of the genome of strain 14-3^T^ has been determined (Tribelli et al., 2012).

Regions of high mountains perennially covered with snow are another abundant source of psychrotrophs. The mean temperature above 2000 m on Changbai Mountain is 3.3°C (Liu 1997). We report here the isolation of a *P*. *mandelii* strain designated CBS-1, a facultative psychrotroph, from soil located at an altitude of 2183 m. Species identification was based on sequence analysis of 16S rRNA and the results of the Biolog GN assay. Strain CBS-1 grew to high density (29.3 g/L of CDW at the stationary phase) and robustly produced PHB (76.1% CDW) at 20°C. The yields of PHB produced at 15, 20, and 25°C were significantly different. The yield of PHB produced at 20°C was significantly higher than at 15°C (*P* < 0.001) and 25°C (*P* < 0.05).

Compared with a few other *Pseudomonas* strains that produce PHB at a low temperature, strain CBS-1 is more promising for industrial use. For example, strain CBS-1 uses sucrose as its best carbon source; therefore, PHB can be produced more economically than by strain 14-3^T^, which requires the more costly carbon source octanoate (Lopez et al., 2009). Moreover, strain CBS-1 grew to a higher maximum cell density than strain 14-3^T^ (Lopez et al., 2009). Jiang et al. (2008) reported that cultivation for 4 days was required to produce the maximum cell density of strain A2a5 and PHB concentration, in contrast to 48 h for strain CBS-1. Therefore, strain CBS-1 may produce higher yields of PHB at a lower cost. Therefore, strain CBS-1 may produce higher yields of PHB at a lower cost and more efficiently than strain 14-3^T^ and A2a5.

## Authors’ information

Rongpeng Li and Yuji Jiang co-first author.
